# Frankincense preparation promotes formation of inflammation-resolving lipid mediators by manipulating lipoxygenases in human innate immune cells

**DOI:** 10.3389/fphar.2023.1332628

**Published:** 2024-01-04

**Authors:** Vivien Nischang, Finja M. Witt, Friedemann Börner, Mario Gomez, Paul M. Jordan, Oliver Werz

**Affiliations:** ^1^ Department of Pharmaceutical/Medicinal Chemistry, Institute of Pharmacy, Friedrich Schiller University Jena, Jena, Germany; ^2^ Evonik Operations GmbH, Darmstadt, Germany; ^3^ Jena Center for Soft Matter (JCSM), Friedrich Schiller University Jena, Jena, Germany

**Keywords:** frankincense, *Boswellia serrata*, boswellic acid, inflammation, lipid mediators, lipoxygenase

## Abstract

**Introduction:** Frankincense preparations are frequently used as traditional anti-inflammatory remedies in folk medicine with increasing popularity. Boswellic acids (BAs), especially 3-O-acetyl-11-keto-βBA (AKBA), are unique anti-inflammatory principles of frankincense, with multiple pharmacological actions and target proteins. We recently showed that AKBA favorably impacts lipid mediator (LM) networks in innate immune cells, by modulation of lipoxygenase (LOX) activities. Thus, AKBA binds to allosteric sites in 5-LOX, shifting the regiospecificity to a 12/15-lipoxygnating enzyme, and to an analogous site in 15-LOX-1, leading to enzyme activation, which favors specialized pro-resolving mediator (SPM) formation at the expense of leukotriene production.

**Methods:** Here, we investigated Boswellin super® (BSR), a commercially available frankincense extract with ≥30% AKBA, used as remedy that approved efficacy in osteoarthritis trials, for its ability to modulate LM pathways in human monocyte-derived macrophage (MDM) phenotypes, neutrophils, and neutrophil/platelet co-incubations. LM profiling was performed by using targeted ultraperformance liquid chromatography-tandem mass spectrometry (UPLC-MS-MS).

**Results:** BSR concentration-dependently (10–100 μg/ml) suppressed formation of pro-inflammatory 5-LOX products including LTB4 in exotoxin-stimulated M1-MDM and neutrophils, and strongly elevated 12/15-LOX products and SPM in activated M2-MDM and neutrophil/platelet cocultures, starting at 10 μg/mL. Also, BSR (≥10 μg/mL) induced robust 12/15-LOX product and SPM generation in resting M2-MDM, which was further markedly elevated when exogenous docosahexaenoic acid (DHA) and eicosahexaenoic acid (EPA) were supplied, and induced translocation of 15-LOX from a soluble to a particulate locale in M2 MDM.

**Discussion:** We conclude that BSR especially when co-added with DHA and EPA, promotes the LM class switch in innate immune cells from pro-inflammatory to pro-resolving mediators, which might be a plausible mechanism underlying the anti-inflammatory actions of BSR.

## 1 Introduction

Acute inflammation upon tissue perturbation is part of the immune response and is required for removal of harmful stimuli and for regeneration of tissues in order to reinstall homeostasis (Meizlish et al., 2021). Misdirection of inflammatory processes leads to unresolved pathological inflammation associated with many devastating chronic diseases, such as arthritis, atherosclerosis, type 2 diabetes, and Alzheimer’s disease (Tabas and Glass, 2013). Pharmacological strategies to treat these diseases pursue dampening of excessive inflammation mainly by applying glucocorticoids (GCs) and non-steroidal anti-inflammatory drugs (NSAIDs) that block the production of pro-inflammatory mediators, including eicosanoids (Rainsford, 2007). However, these drugs can also impair inflammation resolution by inhibiting enzymes involved in the biosynthesis of anti-inflammatory and pro-resolving lipid mediators (LMs) (Tabas and Glass, 2013; Serhan and Levy, 2018).

Pro-inflammatory LMs include prostaglandins (PG) and leukotrienes (LTs), generated from arachidonic acid (AA) by cyclooxygenase (COX) and 5-lipoxygenase (LOX), respectively (Calder, 2020). The specialized pro-resolving mediators (SPMs), however, are distinct LMs that are mainly formed from the omega-3 fatty acids eicosapentaenoic acid (EPA) and docosahexaenoic acid (DHA), which promote inflammation resolution, tissue regeneration, and facilitate the return to homeostasis (Serhan, 2014; Chiang and Serhan, 2020). SPMs are grouped into AA-derived lipoxins (LXs), EPA-derived resolvins (RvEs), and DHA-derived RvDs, protectins (PDs), and maresins (MaRs) that are all potent immunoresolvents with potential for pharmacotherapy of inflammatory diseases without the resolution-toxic effects of GC and NSAIDS (Tabas and Glass, 2013; Serhan and Levy, 2018). Formation of AA- and DHA-derived SPMs requires two sequential oxygenation steps that involve either 12-/15-LOXs alone to form di-hydroxylated PDs and/or MaRs or 12-/15-LOXs acting in conjunction with 5-LOX to form di-/trihydroxylated RVs and LXs (Chiang and Serhan, 2020).

Currently, no pharmacological treatments are approved to stimulate endogenous SPM biosynthesis for promoting inflammation resolution. However, small molecules were recently discovered that favorably modulate the LM biosynthetic network by shifting the production of LTs toward SPM in activated leukocytes and/or even actively elicit SPM formation (Gerstmeier et al., 2019; Werner et al., 2019; Gilbert et al., 2020; Pace et al., 2021; Kretzer et al., 2022a; Kretzer et al., 2022b; Borner et al., 2023). These compounds act by different mechanisms, invoking a regiospecificity shift of 5-LOX toward the 12/15-lipoxygenating enzyme (Gilbert et al., 2020), activating 15-LOX (Kretzer et al., 2022b; Borner et al., 2023), or interfering with 5-LOX-activating protein (FLAP) (Kretzer et al., 2022a). Among these molecules, the pentacyclic triterpene 3-*O*-acetyl-11-keto-β-boswellic acid (AKBA), found in the gum resin of *Boswellia serrata* (termed frankincense), acts as molecular switch blocking LT formation but stimulating SPM production by allosteric modulation of 5- and 15-LOX isoforms (Gilbert et al., 2020; Gilbert et al., 2021; Borner et al., 2023). Frankincense preparations are frequently used as traditional anti-inflammatory remedies for treatment of arthritis, psoriasis and erythematous eczema, and inflammatory bowel disease, and the multiple mechanisms of boswellic acids (BAs), in particular of AKBA, may account for their anti-inflammatory properties (Abdel-Tawab et al., 2011; Efferth and Oesch, 2022).

AKBA, well-known to inhibit 5-LOX (Abdel-Tawab et al., 2011), is bound to 5-LOX at an allosteric site, thereby promoting a shift in the regiospecificity from 5- to 12/15-lipoxygenation (Gilbert et al., 2020). Moreover, AKBA caused allosteric activation of the 15-LOX-1 connected to robust SPM formation in innate immune cells, especially in M2 macrophages (Borner et al., 2023). Such beneficial features of AKBA on LM networks raise the question of whether commercially available frankincense preparations used in folk medicine exert 15-LOX-1 activation and SPM induction as well, substantiating the pharmacological potential of these remedies for treating inflammatory diseases. Thus, we employed the standardized frankincense extract “Boswellin® Super” (BSR, corresponding to 5-Loxin®), a well-known traditional anti-inflammatory remedy with high AKBA contents of at least 30% (Majeed et al., 2021), to study its impact on LM biosynthetic pathways in human immune cells.

## 2 Materials and methods

### 2.1 Materials

“Boswellin® Super” (corresponding to 5-Loxin®, US Patent publication no.: 2004/0073060A1) was prepared from *Boswellia serrata* Roxb. (Burseraceae) and kindly provided by Sabinsa (Europe GmbH, Langen, Germany), batch number C161599. AvailOm® was kindly provided by Evonik (Darmstadt, Germany). All other fine chemicals and bioreagents were obtained from Sigma-Aldrich (Deisenhofen, Germany), unless stated otherwise.

### 2.2 Isolation and culture of human cells

Monocytes, neutrophils, and platelets were isolated from leukocyte concentrates that were obtained from freshly withdrawn peripheral blood of healthy adult (18–65 years) female and male donors with informed written consent (Institute of Transfusion Medicine, Jena University Hospital, Germany). The experimental protocols were approved by the ethical committee of the Jena University Hospital (Approval No. 5050–01/17) and the experimental procedures were conducted in accordance with the relevant guidelines and regulations. For erythrocyte sedimentation, the leukocyte concentrates were mixed with dextran from *Leuconostoc* spp. (MW ∼40,000, Sigma Aldrich). Then, the supernatants were centrifuged on a lymphocyte separation medium (Histopaque®-1077, Sigma Aldrich). The resulting platelet-enriched plasma (top layer) was diluted with PBS pH 5.9 (4:1 *v/v*) and centrifuged (2,100 × g, 15 min, room temperature). The pelleted platelets were resuspended in a 1:1 *v/v* mixture of PBS pH 5.9 and NaCl solution (0.9% m/v), washed two more times, and resuspended in PBS pH 7.4 containing CaCl_2_ (1 mM). The remaining erythrocytes in the pelleted neutrophils were lysed with water (40 s), and neutrophils were washed in PBS pH 7.4 and finally resuspended in PBS pH 7.4 containing CaCl_2_ (1 mM). The peripheral blood mononuclear cell fraction (middle layer) was seeded in RPMI 1640 (Sigma-Aldrich), supplemented with 10% (v/v) heat-inactivated fetal calf serum (FCS), 100 U/mL penicillin, and 100 μg/mL streptomycin in tissue culture flasks (Greiner Bio-one, Frickenhausen, Germany), and kept for 1.5 h at 37°C and 5% CO_2_ to adhere monocytes. For differentiation of monocytes to macrophages using GM-CSF (MDM_GM-CSF_) or M-CSF (MDM_M-CSF_) and further polarization toward M1-MDM (LPS/IFN-γ) or M2-MDM (IL-4), published procedures were used as described in Werz et al. (2018).

### 2.3 Cell viability and cell integrity analysis using the MTT assay and LDH release assay

M1- or M2-MDMs (10^5^/mL) were treated in RPMI 1640 containing 10% (v/v) FCS, 100 U/mL penicillin, and 100 μg/mL streptomycin with AKBA, BSR, or 0.1% vehicle (DMSO) for 180 min; 1% Triton X-100 was used as the positive control.

For analysis of cell viability, cells were incubated with 3-(4,5-dimethylthiazol-2-yl)-2,5-diphenyltetrazolium bromide (MTT, 5 mg/mL, 20 μL; Sigma-Aldrich, Munich, Germany) for 2–3 h at 37°C and 5% CO_2_ in the dark. The formazan product was solubilized with sodium dodecyl sulfate (SDS, 10% in 20 mM HCl), and the absorbance was monitored at 570 nm using a Multiskan Spectrum microplate reader (Thermo Fisher Scientific, Schwerte, Germany).

For analysis of cell integrity, the release of lactate dehydrogenase (LDH) was assessed by CytoTox 96® Non-Radioactive Cytotoxicity assay according to the manufacturer’s (Promega, Mannheim, Germany) instructions. The cells were centrifuged at 400 × g (5 min, 4°C), and the supernatants were diluted to appropriate LDH concentrations. The absorbance was monitored at 490 nm using a NOVOstar microplate reader (BMG LABTECH GmbH, Offenburg, Germany). Cell integrity was calculated according to the manufacturer’s guidelines.

### 2.4 Incubation for LM formation and LM metabololipidomics using UPLC-MS-MS

To study the effects of the BSR on LM formation, neutrophils (5 × 10^6^/mL), platelets (1 × 10^8^/mL), M1- or M2-MDM (2 × 10^6^/mL), and co-cultures of neutrophils (5 × 10^6^/mL) and platelets (1 × 10^8^/mL) were incubated in PBS containing 1 mM CaCl_2_ with BSR or vehicle (ethanol 0.2%) for 30 min, prior to the addition of SACM (1%) for 90 min, or for 180 min in the absence of any stimulus, at 37°C and 5% CO_2_. In some experiments, BSR with or without SACM (1%) was added together with 3 μg/mL AvailOm®, and incubations were performed for 90 or 180 min, respectively, at 37°C and 5% CO_2_. The reactions were stopped by the addition of 2 mL of ice-cold methanol containing deuterated LM standards (200 nM d8-5S-HETE, d4-LTB_4_, d5-LXA_4_, d5-RvD2, d4-PGE_2_, and 10 µM d8-AA; Cayman Chemical/Biomol GmbH, Hamburg, Germany). The samples were kept at −20°C for at least 60 min to allow protein precipitation, and LM were extracted as reported before Jordan et al. (2020). LM were analyzed with an ACQUITY™ UPLC system (Waters, Milford, MA, United States) using an ACQUITY UPLC® BEH C18 column (1.7 µm, 2.1 mm × 100 mm; Waters, Eschborn, Germany) and a QTRAP 5500 Mass Spectrometer (ABSciex, Darmstadt, Germany) equipped with a Turbo V™ Source and electrospray ionization as reported before by Werner et al. (2019). For UPLC-MS-MS analysis, the quantification limit was 3 pg/sample and this value was taken to express the fold increase for samples where the LM was below the detection limit. The UPLC-MS-MS chromatograms of standards and biological samples of the SPMs RvD5, RvE4, and LXA_4_ are shown in the Supplementary Material (Supplementary Figure S1).

### 2.5 Subcellular localization of 5-LOX and 15-LOX-1 by immunofluorescence microscopy

M0_M-CSF_ MDM (0.5 × 10^6^ cells), seeded onto glass coverslips, were polarized to M2-MDM for 48 h, washed with PBS pH 7.4, and PBS pH 7.4 containing CaCl_2_ (1 mM) was added. The cells were incubated with the BSR, SACM [1%; as positive control (Jordan et al., 2020)] or vehicle (DMSO; 0.1%) for 90 min at 37°C and 5% CO_2_. The cells were fixed, permeabilized, and analyzed for subcellular localization of FLAP, 5-LOX, and 15-LOX-1 by immunofluorescence microscopy as reported before Jordan et al. (2020).

### 2.6 Statistical analysis

The results are given as means ± standard error of the mean (SEM) of n observations, where n represents the number of experiments with separate donors, performed on different days, as indicated. Analyses of the data were conducted using GraphPad Prism 8 software (San Diego, CA). The two-tailed t-test was used for comparison of two groups. For multiple comparison, one-way analysis of variance (ANOVA) with Dunnett’s or Tukey’s *post hoc* tests were applied as indicated. The criterion for statistical significance is *p* < 0.05.

## 3 Results

### 3.1 Boswellin® Super (BSR) inhibits formation of 5-LOX products but increases the formation of 12/15-LOX products in human MDM

**FIGURE 1:**
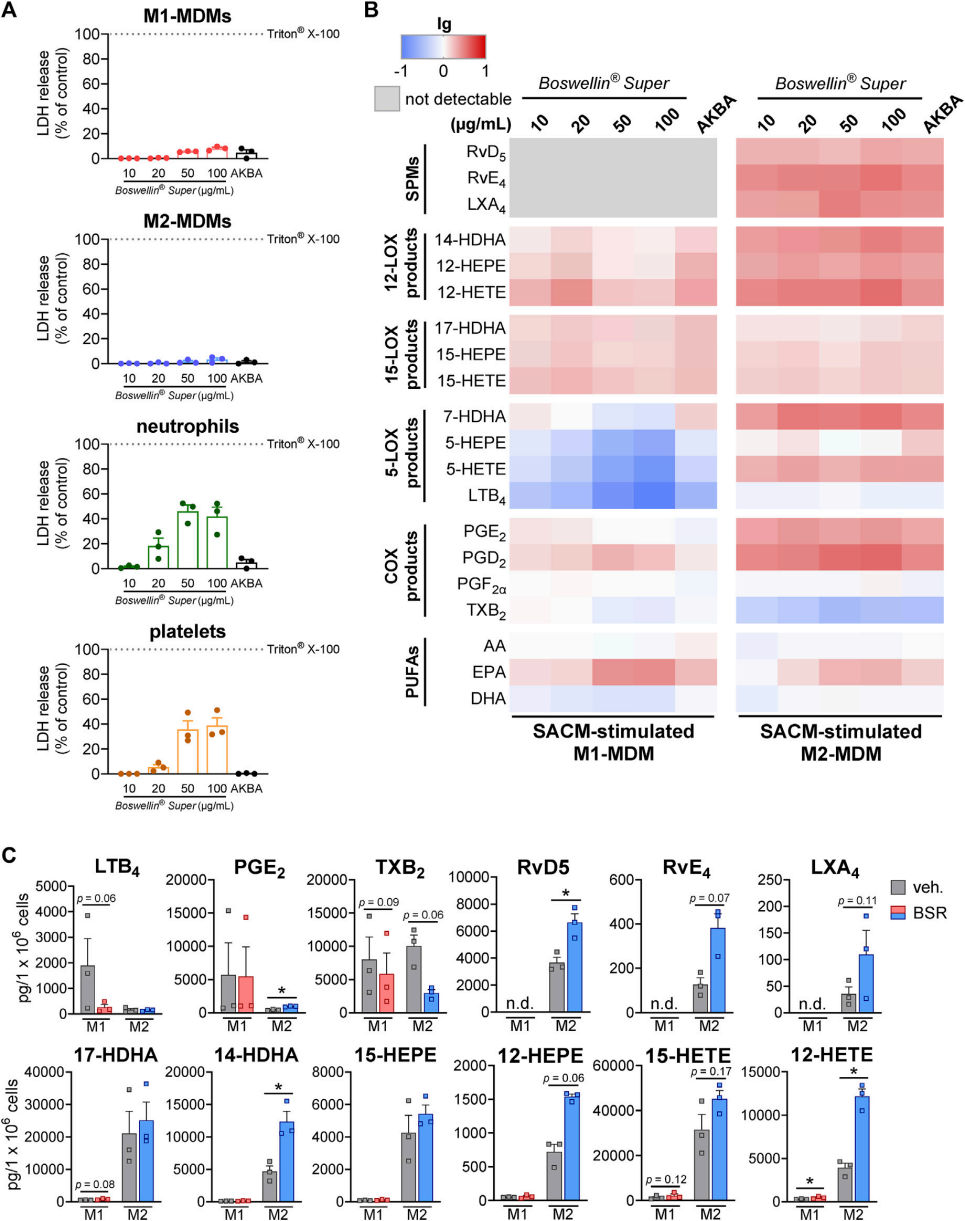
Boswellin® Super (BSR) inhibits formation of 5-LOX products but elevates the formation of 12/15-LOX products in human MDM. **(A)** Impact of Boswellin® Super (BSR) on cell viability of human innate immune cells. LDH release of 1 × 10^6^ M1- and M2-MDMs, 5 × 10^6^ neutrophils, and 5 × 10^8^ platelets after treatment with indicated concentration of BSR or AKBA (10 µM). Triton X-100 was used as a positive control (max. LDH release), and % of control was calculated as per the manufacturer´s guidelines, n = 3 separate donors. **(B,C)** Human M1-or M2-MDMs (10^6^/mL) were pre-incubated with the indicated concentrations of BSR, AKBA (10 µM) or vehicle (0.2% ethanol) for 30 min and then stimulated with 1% SACM for 90 min at 37°C. Formed LM were isolated from the supernatants and analyzed by UPLC-MS/MS. **(B)** Heatmap showing the fold changes (logarithmic scale) in LM formation for BSR or AKBA versus vehicle-pretreated cells stimulated with 1% SACM, *n* = 3, separate donors. **(C)** Data for M1- and M2-MDMs treated with 50 μg/mL BSR or vehicle (veh.), given as pg/10^6^ cells, means ± S.E.M., n = 3, separate donors. For statistical analysis, data were log-transformed, ratio-paired t-test, *p < 0.05, BSR vs. vehicle control.

Previous studies with AKBA to modulate LOX and COX pathways revealed effective and pharmacological relevant concentrations of AKBA in the range of 3–100 µM (Poeckel et al., 2006; Siemoneit et al., 2008; Gilbert et al., 2020; Borner et al., 2023). Thus, we consistently analyzed the effects of BSR at 10, 20, 50 or 100 μg/mL corresponding to approx. 6.5, 13, 33 or 65 μM AKBA, based on the AKBA content in BSR (30%). We first examined the impact of BSR on the membrane integrity of M1- and M2-MDMs, neutrophils, and platelets within 3 h (maximal incubation time with BSR in this study). BSR showed no detrimental effect up to 100 μg/mL for the LDH release of all investigated innate immune cells (Figure 1A). We then analyzed the effect of BSR in modulating LM formation in human exotoxin-stimulated MDM with the M1 and M2 phenotype by employing comprehensive LM metabololipidomics using UPLC-MS/MS. For cell stimulation, we used the *Staphylococcus aureus*-conditioned medium (SACM), containing exotoxins that evoke COX- and 5-LOX-derived LM formation in M1-MDM and robustly elicit the generation of 12/15-LOX products and SPM in M2-MDM (Jordan et al., 2020).

**FIGURE 2:**
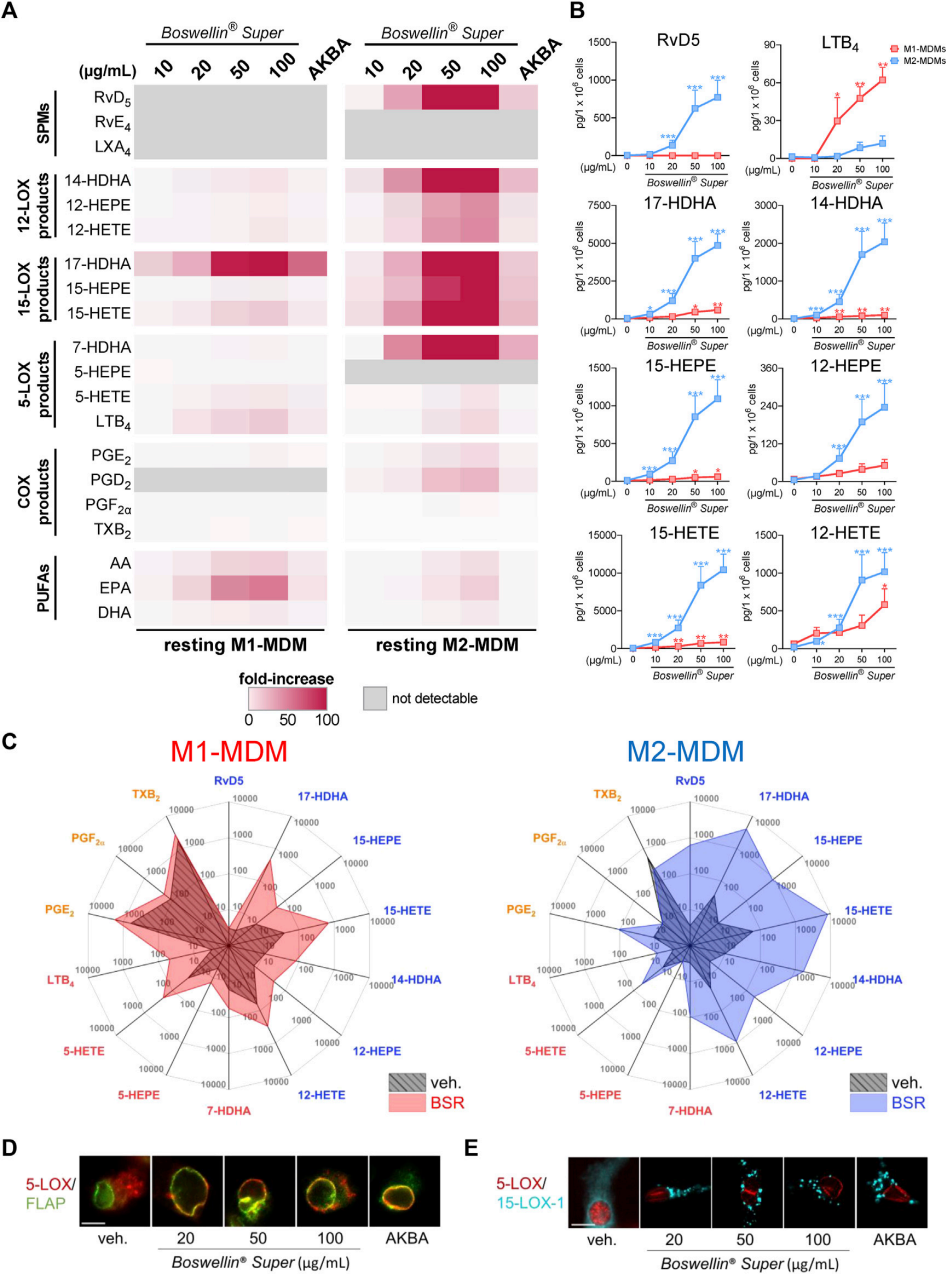
Boswellin® Super (BSR) potently induces formation of 12/15-LOX products in human MDM. **(A,B)** Human M1-or M2-MDMs (10^6^/mL) were incubated with the indicated concentrations of BSR, AKBA (10 µM), or vehicle (0.2% ethanol) for 180 min at 37°C. Formed LM were isolated from the supernatants and analyzed by UPLC-MS/MS. **(A)** Heatmap showing the fold increase in LM formation for BSR- or AKBA- versus vehicle-treated cells, *n* = 3, separate donors. **(B)** Data for M1- and M2-MDMs treated with indicated concentrations of BSR are given as pg/10^6^ cells for M1- (red) and M2-MDM (blue), means ± S.E.M., *n* = 3, separate donors. For statistical analysis, data were log-transformed, one-way analysis of variance (ANOVA) with Dunnett’s multiple comparison test, *p < 0.05, **p < 0.01, and ***p < 0.001 against the vehicle. **(C)** Radar plot showing selected LM (pg per 10^6^ M1- and M2-MDMs) formed by cells after BSR (50 μg/mL) treatment compared to vehicle controls. **(D,E)** Subcellular redistribution of 5-LOX and FLAP in M1-MDMs **(D)** and of 5-LOX and 15-LOX-1 in M2-MDMs **(E)** after treatment with the indicated concentrations of BSR or AKBA (10 µM) for 180 min at 37°C. The cells were fixed, permeabilized, and incubated with antibodies against 5-LOX (red), FLAP (green), and 15-LOX-1 (cyan blue); scale bars, 10 mm. Results shown for a single cell are representative for approximately 100 individual cells analyzed in *n* = 3 independent experiments with separate donors, each.

As expected, and in analogy to AKBA [10 μM, positive control (Borner et al., 2023)], the formation of 5-LOX products (LTB_4_, 5-HETE, and 5-HEPE) in the SACM (1%)-activated M1-MDM within 90 min was concentration-dependently inhibited upon pre-incubation (30 min) with BSR, starting at 10 μg/mL (Figures 1B, C; Table 1). In parallel, products of the 12-LOX (i.e., 14-HDHA, 12-HEPE, and 12-HETE) and the 15-LOX pathway (i.e., 17-HDHA, 15-HEPE, and 15-HETE) were elevated with maximal effects at 20 μg/mL, similar as with 10 µM AKBA. SPM formation was undetectable in M1-MDM, and COX products were hardly affected by BSR and AKBA, except PGD_2_ and PGE_2_, which were found in increased concentration in M2-MDMs; however, their production was moderate compared to formation in M1-MDMs. In addition, release of EPA was increased by BSR and AKBA, while that of AA and DHA was rather diminished (Figure 2B; Table 1).

**TABLE 1:** Modulation of LM pathways in innate immune cells by BSR. M1- and M2-MDMs (10^6^), neutrophils (5 × 10^6^), and platelets (5 × 10^8^) were preincubated with 50 μg/mL BSR or vehicle (0.2% ethanol) for 30 min and then stimulated with 1% SACM for 90 min at 37°C. Formed LM were isolated from the supernatants and analyzed by UPLC-MS/MS and are given in pg (PUFAs in ng) per 10^6^ M1- and M2-MDMs, 5 × 10^6^ neutrophils, and 5 × 10^7^ platelets, *n* = 3.

	pg	M1-MDM			M2-MDM			Neutrophils			Platelets		
		Veh.	BSR	Fold	Veh.	BSR	Fold	Veh.	BSR	Fold	Veh.	BSR	Fold
SPMs	RvD5	≤3	≤3	1.0	3,664 ± 404	6,627 ± 649	1.8	27 ± 13	≤3	0.1	≤3	≤3	1.0
	RvE4	≤3	≤3	1.0	127 ± 30	382 ± 64	3.0	≤3	≤3	1.0	10 ± 3.6	9.1 ± 1.7	0.9
	LXA_4_	≤3	≤3	1.0	35 ± 13	110 ± 45	3.1	≤3	≤3	1.0	≤3	≤3	1.0
12-LOX products	14-HDHA	116 ± 6.2	136 ± 16	1.2	4,675 ± 867	12,337 ± 1,596	2.6	332 ± 68	638 ± 164	1.9	7,678 ± 1,702	6,760 ± 1,472	0.9
	12-HEPE	49 ± 2.5	58 ± 15	1.2	720 ± 115	1,539 ± 37	2.1	240 ± 35	673 ± 209	2.8	4,912 ± 1,395	5,519 ± 1,381	1.1
	12-HETE	300 ± 26	483 ± 83	1.6	3,929 ± 535	12,169 ± 865	3.1	5,740 ± 238	14,173 ± 3,299	2.5	62,466 ± 7,754	87,756 ± 14,716	1.4
15-LOX products	17-HDHA	550 ± 35	806 ± 123	1.5	21,044 ± 6,850	25,084 ± 5,658	1.2	108 ± 59	250 ± 91	2.3	≤3	≤3	1.0
	15-HEPE	80 ± 10	111 ± 22	1.4	4,255 ± 1,081	5,422 ± 552	1.3	21 ± 11	59 ± 27	2.8	≤3	≤3	1.0
	15-HETE	1,346 ± 462	2,188 ± 758	1.6	31,450 ± 6,777	45,198 ± 3,687	1.4	338 ± 138	1,278 ± 458	3.8	40 ± 7.7	51 ± 16	1.3
5-LOX products	7-HDHA	182 ± 29	120 ± 20	0.7	267 ± 32	817 ± 127	3.1	71 ± 12	22 ± 5.1	0.3	≤3	≤3	1.0
	5-HEPE	164 ± 46	41 ± 8.9	0.2	72 ± 12	69 ± 14	0.9	187 ± 20	19 ± 6.9	0.1	≤3	≤3	1.0
	5-HETE	4,654 ± 1,485	884 ± 211	0.2	273 ± 89	538 ± 82	2.0	1,901 ± 346	194 ± 44	0.1	2.9 ± 0.5	3.6 ± 1.0	1.2
	LTB_4_	1,892 ± 1,055	262 ± 113	0.1	164 ± 48	147 ± 22	0.9	2,200 ± 944	53 ± 14	0.0	≤3	≤3	1.0
COX products	PGE_2_	5,701 ± 4,832	5,483 ± 4,447	1.0	423 ± 47	952 ± 43	2.3	98 ± 5.0	422 ± 96	4.3	59 ± 7.2	279 ± 42	4.7
	PGD_2_	166 ± 57	326 ± 172	2.0	205 ± 28	711 ± 121	3.5	12 ± 2.5	57 ± 12	4.6	22 ± 3.2	67 ± 13	3.0
	PGF_2α_	385 ± 116	375 ± 126	1.0	101 ± 26	91 ± 18	0.9	13 ± 0.8	13 ± 4.0	1.0	24 ± 3.2	109 ± 27	4.5
	TXB_2_	7,985 ± 3,404	5,845 ± 3,124	0.7	10,041 ± 1,650	2,981 ± 503	0.3	575 ± 77	792 ± 234	1.4	5,979 ± 654	14,681 ± 3,446	2.5
ng													
PUFAs	AA	1,521 ± 1,375	1,451 ± 51	1.0	955 ± 3,561	872 ± 354	0.9	294 ± 606	719 ± 27	2.4	11 ± 22	19 ± 4.5	1.7
	EPA	390 ± 354	1,027 ± 43	2.6	208 ± 819	386 ± 170	1.9	11 ± 23	106 ± 15	9.2	1.9 ± 3.7	4.1 ± 0.8	2.2
	DHA	926 ± 393	578 ± 64	0.6	430 ± 1,832	439 ± 250	1.0	65 ± 92	124 ± 10	1.9	1.5 ± 2.8	1.3 ± 0.5	0.9

In M2-MDM, BSR at all concentrations as well as 10 μM AKBA, enhanced the formation of all LM in response to 1% SACM within 90 min, except LTB_4_, PGF_2α_, and TXB_2_ that were unaffected or hardly impaired, and also SPM (RvD5, LXA_4_, and RvE4) formation was increased (Figures 2B, C; Table 1). Notably, PUFA release in M2-MDM was modulated by BSR, like in the M1 phenotype, i.e., EPA was elevated, but AA and DHA were unaltered or slightly diminished. Together, BSR mimics the modulatory impact of AKBA on agonist-induced LM production in M1/M2-MDM, with partially even higher effectiveness (at ≥20 μg/mL) for LM upregulation in M2-MDM than 10 μM AKBA, presumably due to the higher AKBA content in BSR (≥13 µM).

### 3.2 Boswellin® Super potently induces formation of 12/15-LOX products in human MDM

Next, we tested whether BSR could act like AKBA (Borner et al., 2023) in inducing LM formation in resting MDM during 3-h incubations. In M1-MDM, BSR concentration-dependently induced the production of all detectable LM, including release of PUFA (again especially of EPA) with superior effectiveness at 50 and 100 μg/mL versus 10 µM AKBA (Figure 2A; Table 2). The striking upregulation of 17-HDHA and to our surprise also of LTB_4_ by BSR, relative to unstimulated controls, is seemingly due to the very low levels of these LM in the resting cells (Figure 2B). In M2-MDM, a similar high effectiveness of BSR was evident for induction of 12/15-LOX products including the SPM RvD5, again much more pronounced by 50 or 100 μg/mL BSR as compared to 10 µM AKBA (Figures 2A, B; Table 2). Induction of the formation of COX- and 5-LOX-derived LM as well as of PUFA release was much less apparent, similar as observed for AKBA, as can be seen in LM radar plots of M1- and M2-MDMs after stimulation with 50 μg/mL BSR (Figure 2C). Analysis of 5-LOX and 15-LOX subcellular redistribution in MDM by IF microscopy showed that exposure to 20–100 μg/mL BSR or 10 µM AKBA induced translocation of both LOXs from soluble to particulate locales within 180 min (Figures 2D, E), indicating activation of these enzymes (Werz et al., 2018; Jordan et al., 2020). In conclusion, BSR mimics the effects of AKBA for eliciting LM formation in resting M1- and M2-MDM, with robust effects on the induction of 12/15-LOX products and RvD5 in M2-MDM, presumably by activation of 15-LOX-1, in analogy to AKBA (Borner et al., 2023).

**TABLE 2:** Induction of LM pathways in innate immune cells by BSR. M1- and M2-MDMs (10^6^) neutrophils (5 × 10^6^), and platelets (5 × 10^8^) were pre-incubated with 50 μg/mL BSR or vehicle (0.2% ethanol) for 180 min at 37°C. Formed LM were isolated from the supernatants and analyzed by UPLC-MS/MS and are given in pg (PUFAs in ng) per 10^6^ M1- and M2-MDMs, 5 × 10^6^ neutrophils, and 5 × 10^7^ platelets, *n* = 3.

	pg	M1-MDM			M2-MDM			Neutrophils			Platelets		
		Veh.	BSR	Fold	Veh.	BSR	Fold	Veh.	BSR	Fold	Veh.	BSR	Fold
SPMs	RvD5	≤3	≤3	1.0	3.6 ± 2.1	625 ± 242	173	≤3	≤3	1.0	≤3	≤3	1.0
	RvE4	≤3	≤3	1.0	≤3	≤3	1.0	≤3	≤3	1.0	≤3	≤3	1.0
	LXA_4_	≤3	≤3	1.0	≤3	≤3	1.0	≤3	≤3	1.0	≤3	≤3	1.0
12-LOX products	14-HDHA	11 ± 6	80 ± 12	7.5	10 ± 4.6	1,706 ± 607	171	8.9 ± 3.5	161 ± 23	18	869 ± 269	6,160 ± 1,144	7.1
	12-HEPE	8 ± 8	39 ± 17	5.0	5.0 ± 2.6	189 ± 72	38	≤3	284 ± 73	95	679 ± 237	7,587 ± 1,577	11
	12-HETE	64 ± 35	307 ± 137	4.8	21 ± 3.5	908 ± 336	44	88 ± 19	6,253 ± 871	71	10,191 ± 3,074	1,07,653 ± 11,637	11
15-LOX products	17-HDHA	≤3	454 ± 73	151	36 ± 18	4,003 ± 1,113	112	≤3	87 ± 32	29	≤3	≤3	1.0
	15-HEPE	9 ± 9.0	50 ± 8.5	5.5	10 ± 5.9	857 ± 273	85	≤3	34 ± 20	11	≤3	≤3	1.0
	15-HETE	38 ± 19	692 ± 99	18	63 ± 10	8,376 ± 2,461	134	16 ± 4.1	840 ± 339	53	22 ± 6.5	32 ± 3.3	1.5
5-LOX products	7-HDHA	17 ± 17	55 ± 3.3	3.3	≤3	92 ± 24	31	≤3	≤3	1.0	≤3	≤3	1.0
	5-HEPE	4.6 ± 2.0	14 ± 1.5	3.0	≤3	≤3	1.0	≤3	2.3 ± 0.5	0.8	≤3	≤3	1.0
	5-HETE	28 ± 10	211 ± 41	7.6	9.3 ± 3.1	52 ± 10	5.6	19 ± 4.7	54 ± 16	2.8	3.1 ± 0.7	3.0 ± 0.5	1.0
	LTB_4_	≤3	48 ± 9.2	16	≤3	8.4 ± 4.3	2.8	4.5 ± 1.2	6.5 ± 0.7	1.4	≤3	≤3	1.0
COX products	PGE_2_	620 ± 440	1,762 ± 1,367	2.8	11 ± 2.9	112 ± 35	10	9.1 ± 1.6	384 ± 107	42	32 ± 6.4	349 ± 24	11
	PGD_2_	≤3	≤3	1.0	5.9 ± 3.2	150 ± 47	25	≤3	47 ± 12.2	16	9.5 ± 1.2	70 ± 6.4	7.4
	PGF_2α_	118 ± 30	195 ± 41	1.7	9.4 ± 3.2	9.3 ± 2.3	1.0	≤3	11 ± 2.7	3.7	12 ± 0.8	124 ± 17	10
	TXB_2_	1,734 ± 511	2,653 ± 875	1.5	545 ± 186	222 ± 31	0.4	149 ± 43	745 ± 195	5.0	4,498 ± 285	15,434 ± 2,673	3.4
ng													
PUFAs	AA	28 ± 5.1	472 ± 102	17	14 ± 1.9	112 ± 41	8.0	11 ± 2.1	190 ± 31	18	3.7 ± 0.5	11 ± 1.8	2.9
	EPA	7.0 ± 0.6	354 ± 60	51	3.6 ± 1.2	39 ± 12	11	0.6 ± 0.1	32 ± 4.8	56	0.5 ± 0.1	2.6 ± 0.3	5.0
	DHA	38 ± 10	248 ± 51	6.5	12 ± 2	64 ± 26	5.2	2.6 ± 0.5	14 ± 2.4	5.5	1.0 ± 0.2	1.0 ± 0.5	1.0

### 3.3 Impact of Boswellin® Super on the modulation and induction of LM formation in human neutrophils

**FIGURE 3:**
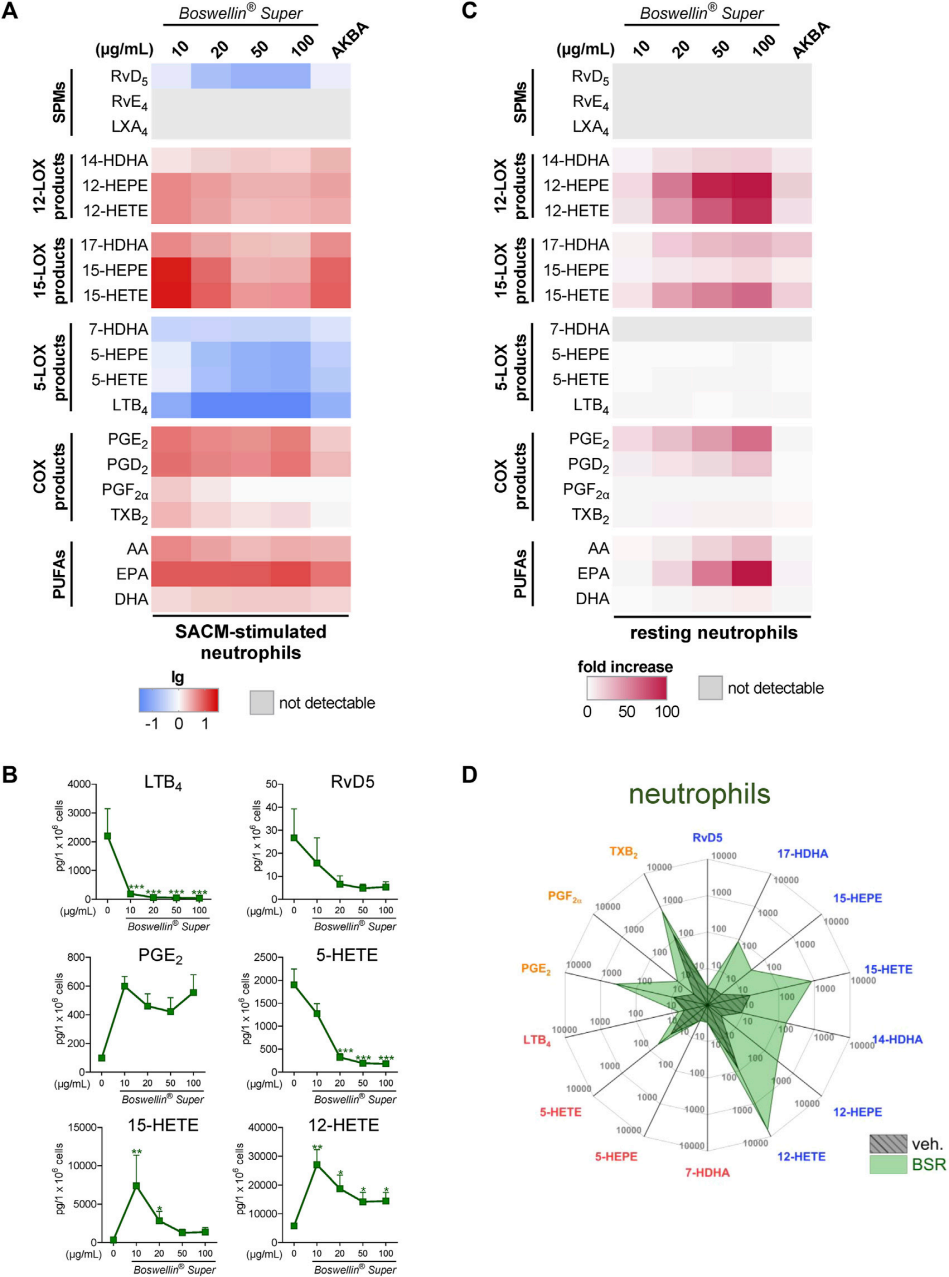
Impact of Boswellin® Super (BSR) on the modulation and induction of LM formation in human neutrophils. **(A,B)** 5 × 10^6^ Human neutrophils/mL were pre-incubated with the indicated concentrations of BSR, AKBA (10 µM), or vehicle (0.2% ethanol) for 30 min and then stimulated with 1% SACM for 90 min at 37°C. Formed LM were isolated from the supernatants and analyzed by UPLC-MS/MS. **(A)** Heatmap showing the fold changes (logarithmic scale) in LM formation for BSR- or AKBA-versus vehicle-pretreated cells stimulated with 1% SACM, *n* = 3, separate donors. **(B)** Data are given as pg/5 × 10^6^ cells, mean ± S.E.M., n = 3, separate donors. For statistical analysis, data were log-transformed, one-way analysis of variance (ANOVA) with Dunnett’s multiple comparison test, *p < 0.05, **p < 0.01, and ***p < 0.001 against vehicle. **(C,D)** 5 × 10^6^ Human neutrophils/mL were incubated with the indicated concentrations of BSR, AKBA (10 µM), or vehicle (0.2% ethanol) for 180 min at 37°C. Formed LM were isolated from the supernatants by SPE and analyzed by UPLC-MS/MS. **(C)** Heatmap showing the fold increase in LM formation for BSR- or AKBA-versus vehicle-treated cells, *n* = 3, separate donors. **(D)** Radar plot showing selected LM (pg per 10^6^ neutrophils) formed by cells after BSR (50 μg/mL) treatment compared to vehicle controls.

In addition to MDM, neutrophils possess high capacities as innate immune cells to generate LM, especially 5-LOX-derived LTB_4_ (Radmark et al., 2015), and AKBA potently inhibited 5-LOX product formation in human activated neutrophils but elevated the levels of 12/15-LOX products in activated and in resting cells (Borner et al., 2023). In SACM (1%)-activated neutrophils, pre-treatment with BSR (30 min) caused efficient and concentration-dependent inhibition of LTB_4_ and 5-HETE generation along with suppression of other 5-LOX products (5-HEPE and 7-HDHA) within 90 min (Figures 3A, B; Table 1). Surprisingly, also RvD5 formation was suppressed by BSR in this manner, suggesting that in neutrophils, RvD5 generation strongly depends on 5-LOX. In contrast, the concentrations of 12/15-LOX products, COX products, and PUFA were all elevated, however, inconsistent with respect to the BSR concentration (Figures 3A, B; Table 1). In fact, it appears that 10 μg/mL BSR is most efficient, especially for 12/15-LOX product formation. Like in M2-MDM, BSR caused robust and concentration-dependent induction of 12/15-LOX products in resting neutrophils, and PGE_2_, PGD_2_, and PUFA release, especially by EPA, was markedly induced, more strikingly than by AKBA (Figures 3C, D; Table 2). 5-LOX product formation was not considerably elicited in resting neutrophils.

### 3.4 Impact of Boswellin® Super on the modulation and induction of transcellular LM formation in co-incubations of human neutrophils and platelets

**FIGURE 4:**
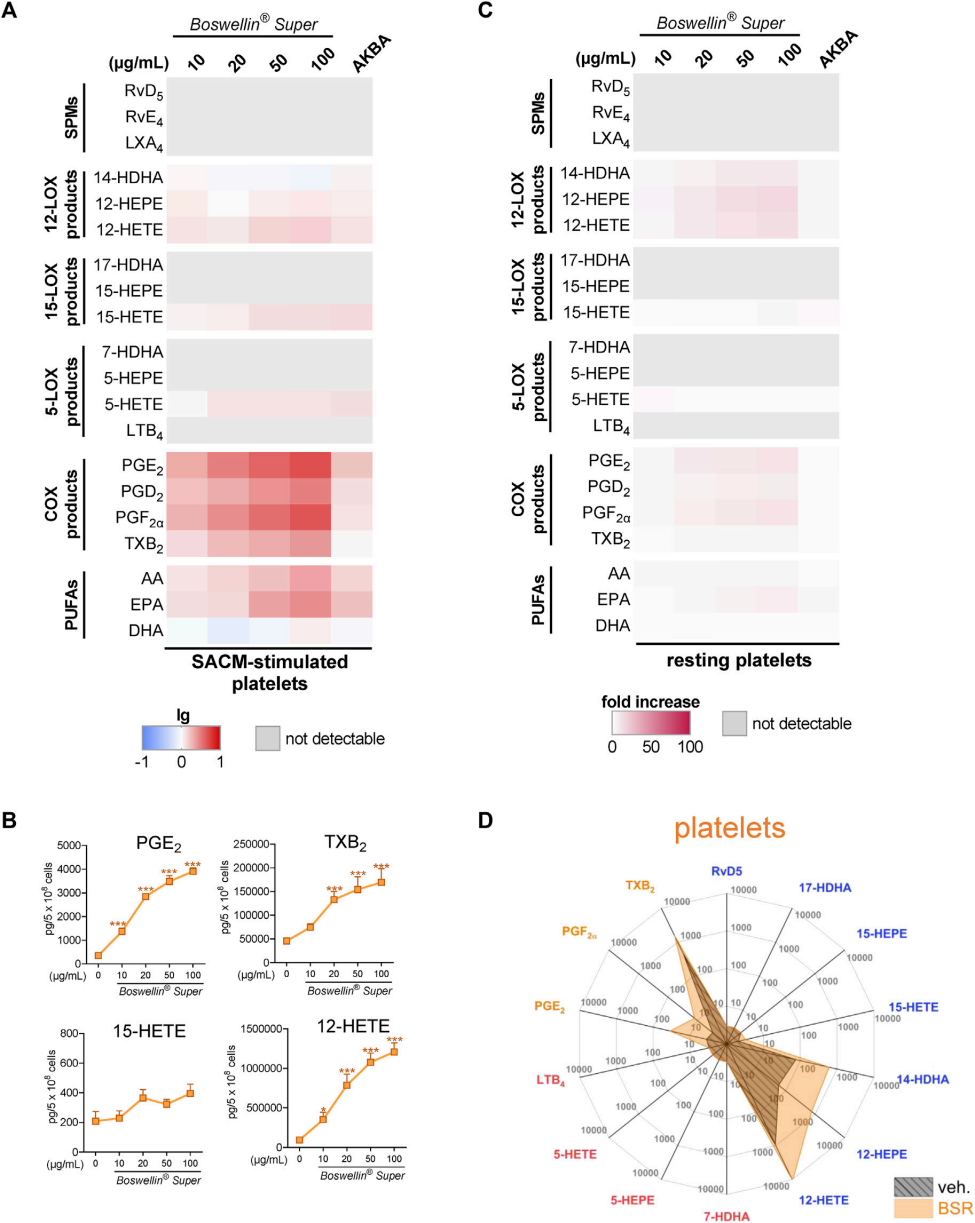
Impact of Boswellin® Super (BSR) on the modulation and induction of LM formation in human platelets. **(A,B)** 5 × 10^8^ Human platelets were pre-incubated with indicated concentrations of BSR, AKBA (10 µM), or vehicle (0.1% ethanol) for 30 min and then stimulated with 1% SACM for 90 min at 37°C. Formed LM were isolated from the supernatants by SPE and analyzed by UPLC-MS/MS. **(A)** Heatmap showing the log-fold changes in LM formation for BSR- or AKBA- versus vehicle-pretreated cells stimulated with 1% SACM, *n* = 3, separate donors. **(B)** Data are given as pg/5 × 10^8^ cells in line charts (orange) with mean ± S.E.M., *n* = 3, separate donors. For statistical analysis, data were log-transformed, one-way analysis of variance (ANOVA) with Dunnett’s multiple comparison test, *p < 0.05; ***p < 0.001 against vehicle. **(C,D)** 5 × 10^8^ Human platelets were incubated with indicated concentrations of BSR, AKBA (10 µM), or vehicle (0.1% ethanol) for 180 min at 37°C. Formed LM were isolated from the supernatants by SPE and analyzed by UPLC-MS/MS. **(C)** Heatmap showing the fold increase in LM formation for BSR- or AKBA- versus vehicle-treated cells, *n* = 3, separate donors. **(D)** Radar plot showing pg of 5 × 10^6^ platelets for selected LM formed by cells after BSR (50 μg/mL) treatment compared to vehicle controls.

Platelets can act in conjunction with neutrophils to enhance LM formation by transcellular metabolism by providing free AA or p12-LOX-derived monohydroxylated LM to neutrophils for generating LTB_4_ and LXA_4_, respectively (Romano and Serhan, 1992; Romano et al., 1993; Capra et al., 2015). Moreover, the 5-LOX/12-LOX product 5,12-diHETE is tremendously formed in such co-cultures and, thus, could not be separated by our UPLC-MS/MS system from LTB_4_ and is therefore given as 5,12-diHETE/LTB_4_. We first investigated how BSR affects LM formation in platelets alone, addressing resting or SACM-activated cells. Due to the marked abundance of p12-LOX and COX-1/TXAS, platelets produced substantial amounts of 14-HDHA, 12-HEPE, and 12-HETE, as well as of PGs, especially TXB_2_. Pre-treatment of platelets with BSR for 30 min prior to stimulation with 1% SACM (90 min) resulted in strong and concentration-dependent elevation of all COX products, 12-HETE and 15-HETE along with AA and EPA release, while DHA and other LOX products, including SPM, were not or only moderately increased (Figures 4A, B; Table 1). AKBA gave a similar pattern, with inferior elevation of COX products (Figures 4A, B). Similarly, BSR induced mainly COX and 12-LOX products as well as AA/EPA release in resting platelets within 3 h, seemingly more pronounced compared to AKBA (Figures 4C, D; Table 2). It should be noted that compared to M1-/M2-MDM and neutrophils, the magnitude of increase of LM products and PUFA release in platelets was less striking.

**FIGURE 5:**
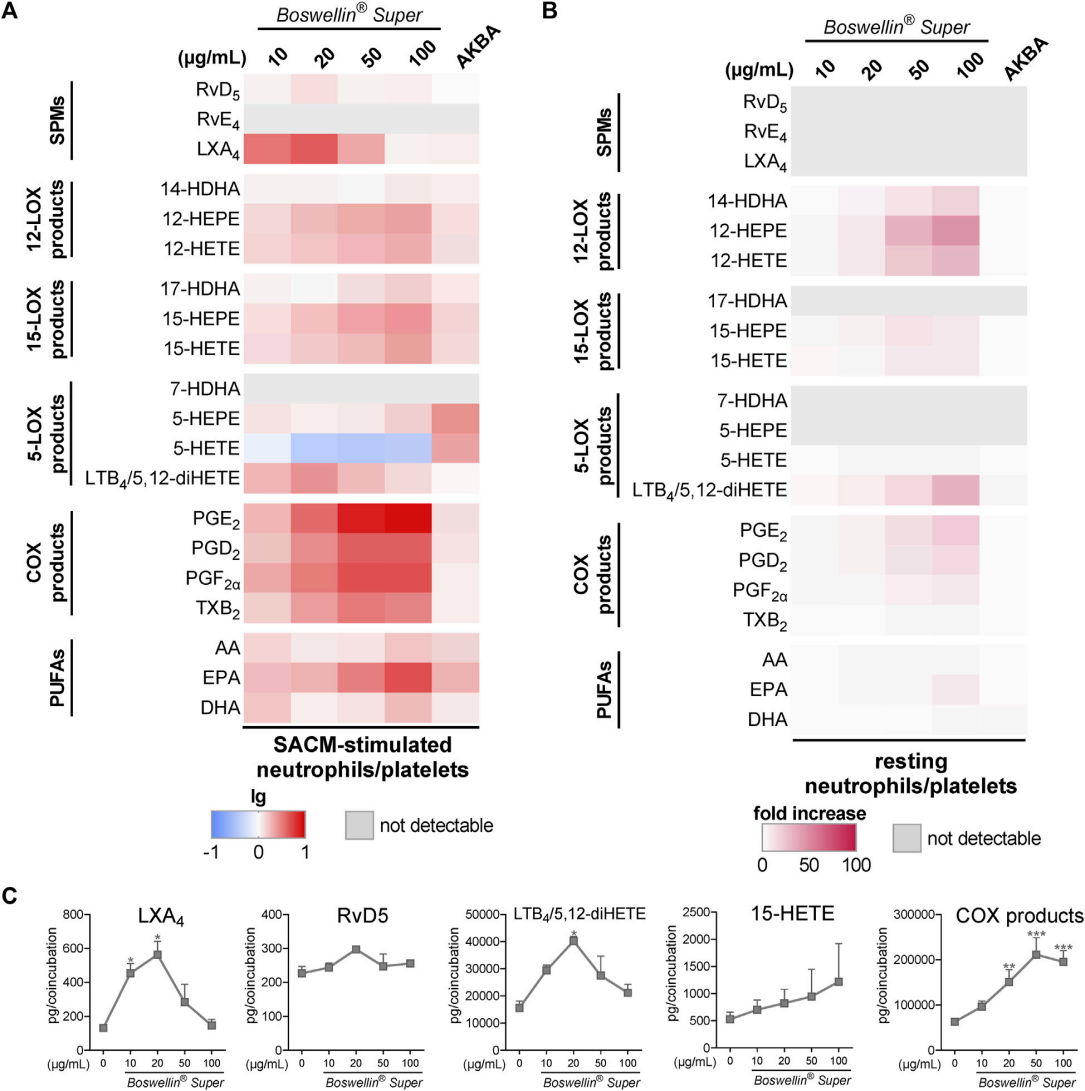
Impact of Boswellin® Super (BSR) on the modulation and induction of transcellular LM formation in co-incubations of human neutrophils and platelets. Co-incubations of neutrophils and platelets (10^7^ and 25 × 10^7^ cells/mL, respectively) were pretreated with the indicated concentrations of BSR, AKBA (10 µM), or vehicle (0.2% ethanol) for 30 min and then stimulated with 1% SACM for 90 min at 37°C. Formed LM were isolated from the supernatants and analyzed by UPLC-MS/MS. **(A)** Heatmap showing the fold changes in LM formation (logarithmic scale) for BSR- or AKBA-versus vehicle-pretreated cells stimulated with 1% SACM, *n* = 3, separate donors. **(B,C)** Co-incubations of neutrophils and platelets (10^7^ and 25 × 10^7^ cells/mL, respectively) with the indicated concentrations of BSR, AKBA (10 µM), or vehicle (0.2% ethanol) for 180 min at 37°C. Formed LM were isolated from the supernatants and analyzed by UPLC-MS/MS. **(B)** Heatmap showing the fold increase in LM formation for BSR- or AKBA- versus vehicle-treated cells, *n* = 3, separate donors. **(C)** Data are given as pg/co-incubation, means ± S.E.M., *n* = 3, separate donors. For statistical analysis, data were log-transformed, one-way analysis of variance (ANOVA) with Dunnett’s multiple comparison test, *p < 0.05, **p < 0.01, and ***p < 0.001 against vehicle.

Pretreatment (30 min) of neutrophil/platelet coincubations with BSR prior to stimulation with 1% SACM, concentration-dependently elevated the formation of all COX-derived LM within 90 min, much more prominent than 10 μM AKBA, even at 10 μg/mL (corresponding to 6.5 µM AKBA) (Figure 5A). In addition, the generation of 12/15-LOX products and PUFA release (i.e., EPA) was strongly elevated by BSR, but not for all LM in a concentration-dependent manner. Especially, LXA_4_ and 5,12-diHETE/LTB_4_ were significantly increased by BSR up to 20 μg/mL, but then again declined up to 100 μg/m BSR, while RvD5 levels remained unaffected (Figure 5C). It should be noted that in contrast to AKBA, BSR strongly and concentration-dependently suppressed 5-HETE formation, implicating inhibitory actions against 5-LOX, although impairment of other 5-LOX products was not immediately apparent (Figure 5A). Nevertheless, inhibition of 5-LOX may explain the bell-shaped concentration–response curve of BSR for LXA_4_ and 5,12-diHETE/LTB_4_ formation visible at ≥50 μg/mL (Figure 5C).

When BSR was added to platelet/neutrophil incubations without any other stimulus for 180 min, again 12/15-LOX and COX products were induced in a concentration-dependent manner, along with the release of PUFAs (Figure 5B). Notably, AKBA failed in this respect, except for some induction of 5,12-diHETE/LTB_4_. 5-LOX products (i.e., 5-HETE and 5-HEPE) were not or hardly produced, and SPMs were not detectable (Figure 5B).

### 3.5 Supplementation of DHA/EPA augments LM formation induced by Boswellin® Super

**FIGURE 6:**
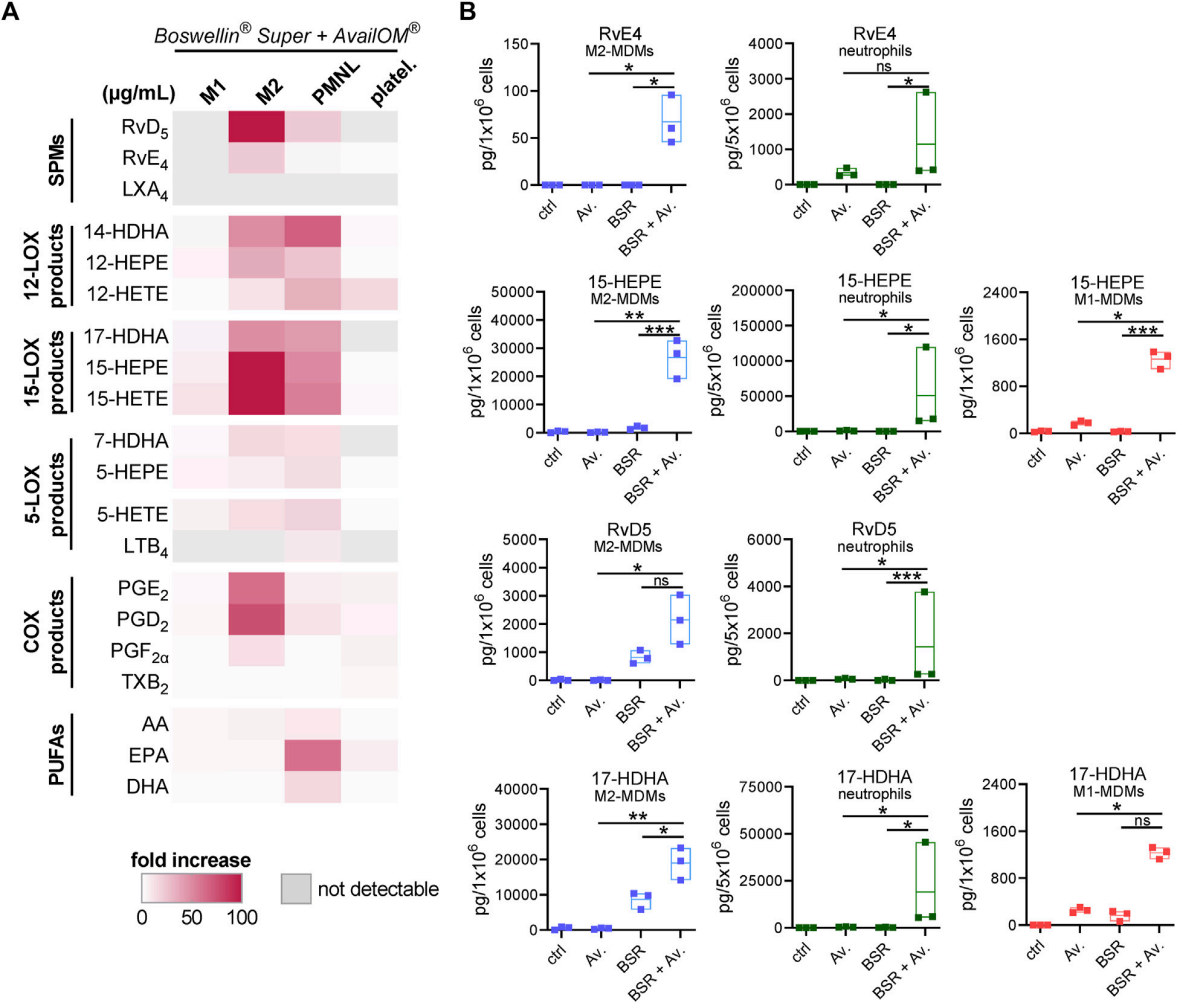
Supplementation of DHA and EPA augments LM formation induced by Boswellin® Super (BSR). **(A,B)** M1- and M2-MDMs (10^6^), neutrophils (5 × 10^6^), and platelets (5 × 10^8^) were incubated with 3 μg/mL AvailOm® (Av.), 50 μg/mL BSR, or BSR and AvailOm® in combination (BSR + Av.) for 180 min at 37°C. **(A)** Heatmap showing the fold increase in LM formation for BSR + Av. against Av. treatment for each cell type, *n* = 3, separate donors. **(B)** Data for selected EPA and DHA products for M1-, M2-MDMs, and neutrophils are given in floating bar charts, means, and single values, *n* = 3, separate donors. For statistical analysis, data were log-transformed, one-way analysis of variance (ANOVA) with Tukey’s multiple comparison test, *p < 0.05, **p < 0.01, and ***p < 0.001.

Induction of LM in intact cells upon stimulation requires two key events: i) release of PUFA as LM substrates and adequate provision to the COX/LOXs (e.g., by FLAP for 5-LOX) and ii) activation of the LOX and their translocation to access the liberated PUFA (Dennis and Norris, 2015; Calder, 2020). We studied if exogenous supplementation of DHA and EPA (applied as AvailOm®, 3 μg/mL) could foster BSR-induced LM formation in MDM, neutrophils, and platelets. First of all, UPLC-MS/MS analysis of AvailOm®, produced from marine oil, confirmed the high content of EPA along with monohydroxylated EPA products (18-, 15-, 12-, 11-, and 5-HEPE) and somewhat less DHA (and 17-, 14-, 13-, 10-, 7-, and 4-HDHA) and rather moderate amounts of AA and its monohydroxylated HETEs (Table 3). As expected, AA-derived LM formed by 5-LOX (i.e., LTB_4_ and 5-HETE) were hardly increased when BSR-stimulated cells were supplemented with AvailOm® in all cell types (Figure 6A). Especially, M2-MDMs and neutrophils efficiently converted supplemented EPA and DHA to the SPMs RvD5 and RvE4 when AvailOm® was in combination with BSR (Figure 6B). Such synergistic effects on 12/15-LOX products and SPM generation were observed in M2-MDM when AKBA was used together with AvailOm® (Borner et al., 2023). In M1-MDM and platelets, the impact of AvailOm® supplementation on BSR-induced LM production was essentially absent (platelets) or less pronounced (M1-MDM), with slight increasing effects of various LOX-mediated monohydroxylated DHA and EPA-derived LM (e.g., 14-HDHA, 12-HEPE, 17-HDHA, 15-HEPE, and 18-HEPE in M1-MDM) (Figure 6A). Taken together, exogenous supply of DHA and EPA blunts the COX-stimulatory effects of BSR and acts efficiently together with BSR to elevate the formation of SPM and their 12/15-LOX-derived monohydroxylated precursors in M2-MDM, but not or only moderate in the other cell types studied.

**TABLE 3:** LM analysis of AvailOM® and BSR**.** **LM of 3 µg AvailOM® and 100 μg BSR, which were used in the cell studies, were measured with UPLC-MS/MS. Data are given in pg**.

		AvailOM®	BSR
EPA	18-HEPE	6.176	—
	15-HEPE	2.175	—
	12-HEPE	2.185	—
	11-HEPE	1.741	—
	5-HEPE	2.380	—
DHA	17-HDHA	1.637	—
	14-HDHA	949	—
	13-HDHA	655	—
	10-HDHA	578	—
	7-HDHA	302	—
	4-HDHA	405	—
AA	15-HETE	174	—
	12-HETE	121	—
	11-HETE	122	—
	5-HETE	78	—

## 4 Discussion

We show that the frankincense extract BSR, containing ≥30% AKBA, mimicked the favorable LOX-modulatory activities of AKBA with respect to inflammation-related LM (Borner et al., 2023) in human innate immune cells. BSR inhibited the pronounced formation of pro-inflammatory 5-LOX products (e.g., LTB_4_) in exotoxin-stimulated M1-MDM and neutrophils, but strongly elevated SPM and 12/15-LOX products in activated M2-MDM, inducing the so-called “LM class switch.” Moreover, like AKBA, BSR elicited robust formation of 12/15-LOX products and SPM generation in resting M2-MDM, which was further markedly elevated when exogenous DHA and EPA were supplied as substrates. Similarly, at low concentrations (≤20 μg/mL), BSR promoted 12/15-LOX product formation, especially LXA_4_ and RvD5 in physiologically relevant neutrophil/platelet co-incubations. These data strongly support the anti-inflammatory and pro-resolving potential of frankincense preparations and their clinical use in treating inflammation-related diseases, especially with co-application of omega-3 PUFAs, represented by AvailOm® in this study, to potentiate *de novo* SPM formation during therapy of inflammatory disorders. However, BSR and AKBA also increased and induced the formation of pro-inflammatory COX products in all cells, but, interestingly, co-addition of AvailOm® seemingly blunted these effects of BSR. Finally, our results imply that diverse LM pathways can be efficiently induced in innate immune cells not only by typical inflammation-related stimuli like bacterial exotoxins, LPS, N-formyl-methionyl-leucyl-phenylalanine, or zymosan (Jordan and Werz, 2021) but also by quite distinct agents (i.e., BSR) that can be operative with relevance for pharmacotherapy.

Frankincense preparations like BSR are frequently used as anti-inflammatory remedies in folk medicine, and several clinical trials support their potential for treatment of osteoarthritis (OA), rheumatoid arthritis, multiple sclerosis, psoriasis and erythematous eczema, inflammatory bowel disease, and certain cancers (Abdel-Tawab et al., 2011; Efferth and Oesch, 2022). In fact, the efficacy of BSR was proven in OA patients (improvement in physical and functional ability, reduction of pain and stiffness) and is used in pharmacotherapy (Majeed et al., 2021). It actually resembles the well-studied 5-Loxin®, a comparable frankincense extract that also contains 30% AKBA, for which a double-blind, randomized, placebo-controlled study was carried out to reveal its efficacy in OA when taking 250 mg of the extract per day (Sengupta et al., 2008).

Although SPMs and related 12/15-LOX have not yet been connected to frankincense-based remedies, previous studies with BSR or 5-Loxin® support the pharmacological relevance of the AKBA-induced LM class switch in rodents. Hence, in a rat OA model, 5-Loxin® (100 and 200 μg/g body weight, p.o.) relieved OA joint pain along with suppression of PGE_2_ and LTB_4_ levels (Shin et al., 2022). Likewise, in rats with collagen-induced arthritis, BSR (40 and 80 μg/g body weight, p.o.) lowered anti-collagen antibodies, cartilage oligomeric matrix protein, C-reactive protein, TNF-α, IL-6, nitric oxide, PGE_2_, and COX-2 levels, along with reduction in arthritic index, paw volume, and joint inflammation (Majeed et al., 2021). In our study, we achieved significant LM modulation with BSR starting at 10 up to 100 μg/mL, which are pharmacologically relevant concentrations, as BSR/5-Loxin® were efficacious when supplied at comparable or even higher doses (i.e., 40–200 μg/g) (Majeed et al., 2021; Shin et al., 2022), as stated above. In light of the effects of BSR/5-Loxin® on inflammatory mediators and enzymes in these studies on animal diseases, it is interesting that exogenously added SPMs reduced the levels of pro-inflammatory cytokines like TNF-α and IL-6 as well as of MMPs and nitric oxide in comparable arthritis models (Chiang and Serhan, 2020). Therefore, BSR could mediate its anti-inflammatory actions by modulating LM production in innate immune cells, i.e., suppression of pro-inflammatory 5-LOX products but elevation of inflammation-resolving SPM.

Our study with human innate immune cells was designed to investigate: a) the modulatory impact of BSR on LM formation evoked by bacterial exotoxins and b) the induction of LM production in resting cells. The *S. aureus* exotoxins α-hemolysin and phenol-soluble modulins contained in the SACM potently induce LM formation in human MDM and neutrophils, respectively (Romp et al., 2019; Jordan et al., 2020), as they fulfill the requirements for cellular production of LM, namely, liberation of free PUFAs by PLA_2_s, LOX activation, and LOX translocation for assembly of LM biosynthetic enzymes at defined subcellular membrane compartments (Radmark et al., 2015; Astudillo et al., 2019; Calder, 2020). However, still, in such exotoxin-challenged cells, BSR or AKBA further elevated the substantial 12/15-LOX product formation, in line with our previous findings (Borner et al., 2023). Since PUFA release was increased by BSR as well, this may contribute to elevated LOX product formation. In particular, EPA was most strikingly affected by BSR in all cell types for yet unknown reasons. Note that AA and EPA are proposed to be provided by the same enzyme, i.e., cPLA_2_, for LM formation, while DHA may be supplied by a different PL, likely an sPLA_2_ (Astudillo et al., 2019). Surprisingly, AA- and DHA-derived 12/15-LOX products increased in the same manner as EPA-derived LM (i.e., 12-HEPE or 15-HEPE) due to the impact of BSR, implying that alternative stimulatory mechanisms, namely, allosteric activation of 15-LOX (Borner et al., 2023) or modulation of 5-LOX´s regiospecificity (Gilbert et al., 2020), are operative. In fact, BSR still elevated 12/15-LOX product formation in M2-MDM when exogenous DHA/EPA were added, where the ample substrate supply would compensate stimulatory effects due to PUFA release.

BSR elicited LM production in all cell types studied, implying that it acts as a stimulus for these cells to induce PUFA release, LOX activation, and LOX subcellular redistribution. Such effects were observed for AKBA too (Borner et al., 2023), which might be causative for the actions of BSR but also for the pentacyclic triterpene celastrol (Pace et al., 2021), for two natural chalcones (Kretzer et al., 2022b), and for the synthetic FLAP antagonist BRP-201 (Kretzer et al., 2022a). These compounds may share common modes of action, e.g., the direct allosteric activation at the C2-like domain of the PLA_2_s and LOXs that are in charge of LM production (Gilbert et al., 2020; Pace et al., 2021; Borner et al., 2023), in line with our present findings with BSR, where AKBA may represent the active principle in this respect. Activation of PLA_2_ and LOX upon cell stimulation is mirrored by the translocation of these enzymes from a soluble to a membranous compartment, where cPLA_2_ and 5-LOX translocate to the nuclear envelope (Radmark et al., 2015; Astudillo et al., 2019), while 15-LOX-1 moves to yet unidentified membranous structures within the cytosol, depending on the cell type (Brinckmann et al., 1998; Werz et al., 2018). BSR as well as AKBA (Gilbert et al., 2020; Borner et al., 2023) induced such LOX subcellular redistribution patterns in M1-/M2-MDM and thus have been classified as LOX activators and inducers of LM product formation, like bacterial exotoxins (Jordan et al., 2020).

Many previous studies revealed AKBA and AKBA-containing frankincense extracts as efficient inhibitors of cellular 5-LOX activity (Poeckel and Werz, 2006; Abdel-Tawab et al., 2011) and also found that BSR blocked 5-LOX product formation, especially in M1-MDM and neutrophils where 5-LOX activity was prominent. Intriguingly, all detected SPMs formed in BSR-activated neutrophils, i.e., RvD5, LXA_4_, and RvE4, require 5-LOX activity (Chiang and Serhan, 2020). Our data suggest that at low concentrations, BSR and AKBA may activate not only 15-LOX but also 5-LOX, but not so at higher concentrations where 5-LOX is inhibited. This may explain why in neutrophil/platelet co-incubations, LXA_4_ and LTB_4_/5,12-diHETE are elevated at low (≤20 μg/mL) but repressed at higher BSR concentrations (50 and 100 μg/mL). Moreover, since AKBA can alter the regiospecificity of 5-LOX, switching toward 12/15-lipoxygenation (Gilbert et al., 2020), the elevated 12/15-LOX products may originate from manipulated 5-LOX. In M2-MDM, however, with lower 5-LOX and FLAP but high 15-LOX-1 expression (Werz et al., 2018; Werner et al., 2019), BSR increased RvD5, LXA_4_, and RvE4 formation and caused even stronger increase in 12/15-LOX product levels, suggesting that 15-LOX-1 activation is the crucial biosynthetic step, and thus 15-LOX-1 is the relevant target of AKBA under these conditions.

Intriguingly, BSR also elevated the levels of AA-derived COX products, especially of PGE_2_ and PGD_2_ in exotoxin-activated MDM, neutrophils, platelets, and neutrophil/platelet co-incubations, and to a minor extent also in resting cells, without consistently increasing free AA levels. Supplementation of AvailOm® (which contains some AA) impaired the stimulatory impact of BSR for COX product formation throughout, indicating that BSR acts by fostering substrate supply. Actually, increased COX product formation by BSR was unexpected as AKBA can inhibit COX enzymes (Abdel-Tawab et al., 2011). Any post-translational stimulatory mechanisms of COX activities that could be governed by BSR/AKBA are not known; further studies are needed to resolve these COX-stimulatory actions. Although COX products, i.e., PGE_2_, are generally considered pro-inflammatory, which they certainly are in the early phase of inflammation, in the resolution phase, PGE_2_ and PGD_2_ have pro-resolving functions (Levy et al., 2001). In this respect, elevated PGE_2_ and PGD_2_ levels due to BSR may also possess favorable features for promoting inflammation resolution.

The multiple mechanisms and target interactions of AKBA and related BAs include inhibition of 5-LOX, platelet-type 12-LOX, COX isoforms, microsomal PGE_2_ synthase-1, the NFκB pathway, cathepsin G, elastase, MMP3, and LL-37 as well as modulation of Ca^2+^, TGFβ/SMAD, Akt, and MAPK signaling, and ROS formation (Abdel-Tawab et al., 2011; Efferth and Oesch, 2022), which are eventually responsible for the anti-inflammatory efficacy of frankincense (Poeckel and Werz, 2006; Efferth and Oesch, 2022). Moreover, besides AKBA, frankincense extracts (including BSR and 5-Loxin®) contain other pharmacological relevant BAs (e.g., β-BA, A-β-BA, and KBA), tirucallic acids, roburic acids, and lupeolic acids with even higher potencies than AKBA (Verhoff et al., 2014). The more pronounced effectiveness of BSR at 50 and 100 μg/mL on LM formation versus 10 µM AKBA is most probably due, at least in MDM and neutrophils, to the consequently higher AKBA contents in BSR, with calculated AKBA levels of 33 and 65 µM in the incubations, respectively. Nevertheless, especially in platelets and neutrophil/platelet co-incubations, the BSR at 10 μg/mL (corresponding to 6.5 µM AKBA) was more effective than 10 μM AKBA, suggesting that other BAs or ingredients in the BSR are operative. In fact, β-BA outperformed AKBA in inducing AA release and 12-LOX product formation in human platelets (Poeckel et al., 2006), while β-BA failed to induce LM formation in human MDM (Borner et al., 2023). Platelets may provide free AA or monohydroxylated LM precursors generated by p12-LOX to neutrophils with abundant 5-LOX for generating LTB_4_ and LXA_4_, respectively (Romano and Serhan, 1992; Romano et al., 1993; Capra et al., 2015), as confirmed in our study by the marked formation of LTB_4_/5,12-diHETE and LXA_4_ in response to BSR.

Together, AKBA-containing BSR at pharmacological relevant concentrations promotes the LM class switch in innate immune cells from pro-inflammatory LTs toward pro-resolving SPM. Many of these LM-modulatory actions of BSR might be caused by the major ingredient AKBA, which differentially impacts the key biosynthetic enzymes 5-LOX and 15-LOX-1 (Gilbert et al., 2020; Borner et al., 2023), but it appears that additional components of BSR may contribute to its overall beneficial impact on LM biosynthesis. The additive or even synergistic effects of AvailOm® in combination with BSR on SPM formation has important implications for pharmacotherapy, as BSR/AvailOm® co-application may enhance SPM levels *in vivo* in diseased patients. Both frankincense and DHA/EPA supplements are typical nutraceuticals taken by patients with inflammatory disorders that are associated with low SPM levels; endogenous elevation of SPM, potentially by combined intake of the two nutritional supplements, is inferred as a favorable therapeutic strategy for better resolving inflammatory diseases. In fact, a *Boswellia serrata* extract/AvailOm® combination product was recently shown to improve pain and function of the knee in subjects >40 years with persistent knee pain (Perez-Pinero et al., 2023).

## Data Availability

The original contributions presented in the study are included in the article/Supplementary Material; further inquiries can be directed to the corresponding authors.
