# Supplementary material for: Frankincense preparation promotes formation of inflammation-resolving lipid mediators by manipulating lipoxygenases in human innate immune cells
Source: Front Pharmacol. 2024 Jan 4;14:1332628. doi: 10.3389/fphar.2023.1332628 (PMC10794731; doi:10.3389/fphar.2023.1332628)
Supplement: Supplementary file 1 [file DataSheet1.DOCX]

Supplementary Material

Frankincense preparation promotes formation of inflammation-resolving lipid mediators by manipulating lipoxygenases in human innate immune cells

Vivien Nischang, Finja M. Witt, Friedemann Börner, Mario Gomez, Paul M. Jordan^*^_,_ Oliver Werz^*^

*** Correspondence:**Paul M. Jordan and Oliver Werz
[paul.jordan@uni-jena.de](mailto:paul.jordan@uni-jena.de); oliver.werz@uni-jena.de

## Supplementary Figure

**Supplementary Figure 1.** **UPLC-MS/MS chromatograms that exemplify the identification and quantification of lipid mediators.** Selective ion monitoring (SIM) of RvD5 for Q1:359.2 and Q3:119.1, of RvE4 for Q1:333.0 and Q3:199.0, and of LXA_4_ for Q1:351.2 and Q3:115.1 in the standard mix (STD-mix) in (**A**) and in representatives samples (**B**) of M2-MDMs stimulated with BSR and AvailOm for RvD5, of neutrophils stimulated with BSR and Av. for RvE4, and of neutrophil/platelet coincubations stimulated with SACM 1% for LXA_4_, data are shown in Figures 5 and 6.
